# Effect of the Novel Influenza A (H1N1) Virus in the Human Immune System

**DOI:** 10.1371/journal.pone.0008393

**Published:** 2009-12-23

**Authors:** Evangelos J. Giamarellos-Bourboulis, Maria Raftogiannis, Anastasia Antonopoulou, Fotini Baziaka, Pantelis Koutoukas, Athina Savva, Theodora Kanni, Marianna Georgitsi, Aikaterini Pistiki, Thomas Tsaganos, Nikolaos Pelekanos, Sofia Athanassia, Labrini Galani, Efthymia Giannitsioti, Dimitra Kavatha, Flora Kontopidou, Maria Mouktaroudi, Garyfallia Poulakou, Vissaria Sakka, Periklis Panagopoulos, Antonios Papadopoulos, Kyriaki Kanellakopoulou, Helen Giamarellou

**Affiliations:** 4th Department of Internal Medicine, University of Athens, Medical School, Greece, Athens, Greece; Karolinska Institutet, Sweden

## Abstract

**Background:**

The pandemic by the novel H1N1 virus has created the need to study any probable effects of that infection in the immune system of the host.

**Methodology/Principal Findings:**

Blood was sampled within the first two days of the presentation of signs of infection from 10 healthy volunteers; from 18 cases of flu-like syndrome; and from 31 cases of infection by H1N1 confirmed by reverse RT-PCR. Absolute counts of subtypes of monocytes and of lymphocytes were determined after staining with monoclonal antibodies and analysis by flow cytometry. Peripheral blood mononuclear cells (PBMCs) were isolated from patients and stimulated with various bacterial stimuli. Concentrations of tumour necrosis factor-alpha, interleukin (IL)-1beta, IL-6, IL-18, interferon (FN)-alpha and of IFN-gamma were estimated in supernatants by an enzyme immunoassay. Infection by H1N1 was accompanied by an increase of monocytes. PBMCs of patients evoked strong cytokine production after stimulation with most of bacterial stimuli. Defective cytokine responses were shown in response to stimulation with phytohemagglutin and with heat-killed *Streptococcus pneumoniae*. Adaptive immune responses of H1N1-infected patients were characterized by decreases of CD4-lymphocytes and of B-lymphocytes and by increase of T-regulatory lymphocytes (Tregs).

**Conclusions/Significance:**

Infection by the H1N1 virus is accompanied by a characteristic impairment of the innate immune responses characterized by defective cytokine responses to *S.pneumoniae*. Alterations of the adaptive immune responses are predominated by increase of Tregs. These findings signify a predisposition for pneumococcal infections after infection by H1N1 influenza.

## Introduction

Our world is facing the pandemic of influenza H1N1 viral infection with probable immense effects on daily lives and on the world economy due to the easiness of transmission and to the high virulence of the virus [1], [2]. As of August 30^th^, world health organization (WHO) has reported 254206 laboratory-confirmed cases of infection of H1N1 infection; as of August 2^nd^ 26513 of these cases have occurred in the European continent [3], [4]. These data are considered to be an under-estimation of the situation occurring worldwide due to the existence of cases with a very mild physical course not seeking medical attention. However, a total of 2837 deaths have been reported from the confirmed cases.

The main question that needs to be answered when taking care of a severely ill patient infected by the H1N1 virus is if deterioration of the human host is due to the virus per se or to impairment of the immune system created in the field of infection and to any subsequent predilection for super-infections. Data to provide a clear-cut answer are missing. The present study is aiming to evaluate if infection by the novel H1N1 virus may lead to impairment of the function of the innate and adaptive immune responses of the human host.

## Results

### Demographic Characteristics

A total of 110 patients were screened; 59 were enrolled in the study. Thirty-one patients were infected by H1N1 virus and six developed H1N1-related pneumonia; their mean ± SD age was 28.5±11.6 years; 17 were male and 14 female. Fever and malaise were the predominant symptoms occurring in all patients (100%) followed by cough in seven (22.5%) patients; and diarrhea in three patients (9.8%). Mean ± SD white blood cells were 5708.0±2578.8/µl. No underlying medical history was reported in any patient. Infection convalescence was noted in all.

Eighteen patients were presented with flu-like syndrome; their mean ± age was 39.6±15.2 years; 10 were male and eight female. Fever and malaise were the predominant symptoms occurring in all patients (100%) followed by cough in six (31.6%) patients; and diarrhea in three patients (15.8%). Mean ± SD white blood cells were 8722.2±4817.3/µl. No underlying medical history was reported in any patient. None of these patients developed pneumonia. Infection convalescence was noted in all.

Mean ± SD age of healthy volunteers was 36.3±7.4 years. Mean ± SD white blood cells were 5170.0±1296.2/µl.

### Effects in the Innate Immune System

**Figure 1:**
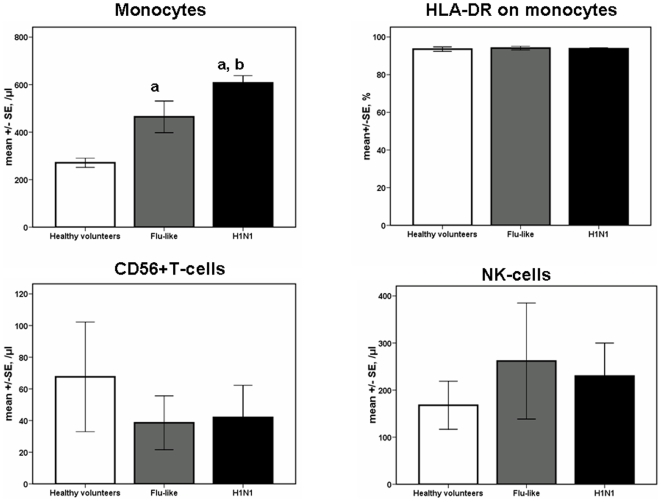
Absolute counts of monocytes, of natural killer T-cell (NKT) and of natural killer (NK) cells. Results refer to 10 healthy volunteers, 18 patients with flu-like syndrome and 31 patients infected by the H1N1 virus. Expression of HLA-DR on monocytes is also shown. a: denotes statistically significant differences compared with healthy volunteers; b: denotes statistically significant differences compared with patients with a flu-like syndrome.

Infection by the H1N1 virus was accompanied by significant increase of monocytes (p<0.0001 compared with healthy volunteers). Co-expression of HLA-DR on monocytes was more than 96% in all patients (Figure 1)

Mean rates of apoptosis of monocytes were 52.4%, 65.5% and 75.5% among healthy volunteers, among patients with flu-like syndrome and among patients infected by the H1N1 virus respectively (p non-significant between groups). Respective mean rates of apoptosis of NKT-cells were 16.6%, 34.3% and 39.1% (p non-significant between groups). Respective mean rates of apoptosis of NK-cells were 19.1%, 27.2% and 23.1% (p non-significant between groups).

**Figure 2:**
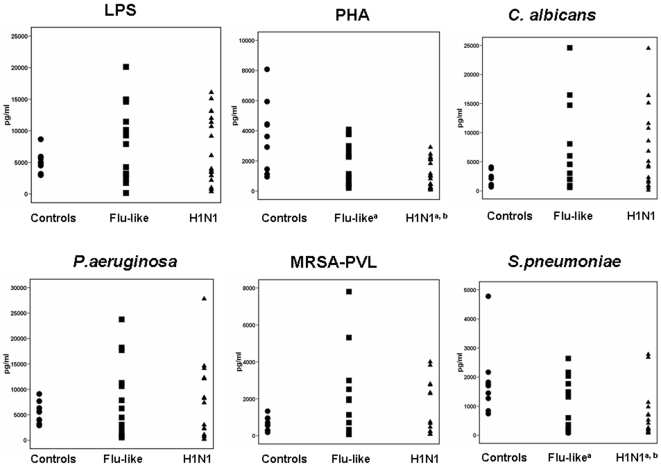
Production of tumour necrosis factor-alpha (TNFα). Peripheral blood mononuclear cells of 10 healthy volunteers, of 18 patients with flu-like syndrome and of 31 patients infected by the H1N1 virus were stimulated with endotoxins (LPS), phytohemagglutin (PHA), and heat-killed isolates of *Candida albicans*, of *Pseudomonas aeruginosa*, of methicillin-resistant *Staphylococcus aureus* producing Panton-Valentine leukocidin (MRSA-PVL) and of penicillin-susceptible *Streptococcus pneumoniae*. Superscript “a” denotes statistically significant differences compared with healthy volunteers; superscript “b” denotes statistically significant differences compared with patients with flu-like syndrome.

Concentrations of TNFα of PBMCs-stimulated supernatants are shown in Figure 2. Patients infected by the H1N1 virus had lower production of TNFα after stimulation with PHA (p<0.0001 compared with healthy volunteers) and heat-killed PSSP (p<0.0001 compared with healthy volunteers).

**Figure 3:**
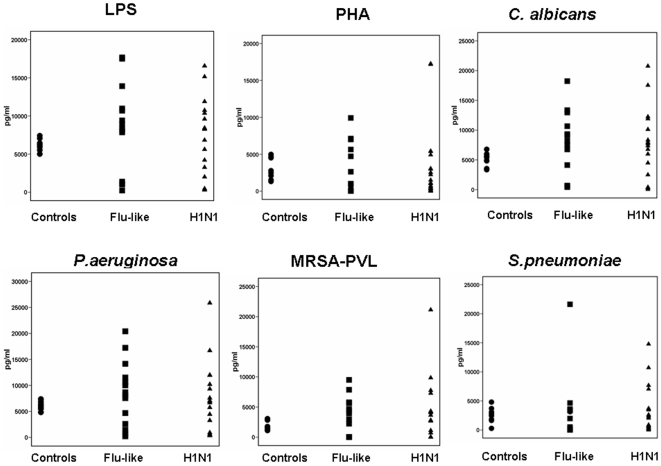
Production of interleukin-1beta (IL-1β). Peripheral blood mononuclear cells of 10 healthy volunteers, of 18 patients with flu-like syndrome and of 31 patients infected by the H1N1 virus were stimulated with endotoxins (LPS), phytohemagglutin (PHA), and heat-killed isolates of *Candida albicans*, of *Pseudomonas aeruginosa*, of methicillin-resistant *Staphylococcus aureus* producing Panton-Valentine leukocidin (MRSA-PVL) and of penicillin-susceptible *Streptococcus pneumoniae*.

Concentrations of IL-1β of PBMCs-stimulated supernatants are shown in Figure 3. No differences were found between healthy volunteers, patients with flu-like syndrome and patients infected by the H1N1 virus.

**Figure 4:**
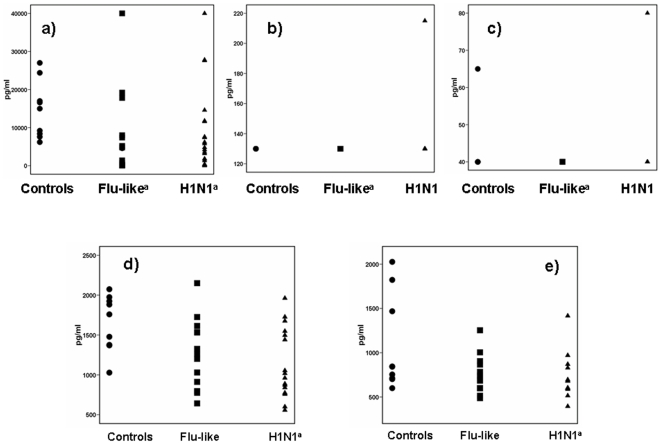
Cytokine production by peripheral blood mononuclear cells of healthy volunteers, of patients with flu-like syndrome and of patients infected by the H1N1. a) interleukin-6 (IL-6) after stimulation with one heat-killed isolate of penicillin-susceptible *Streptococcus pneumoniae*; b) IL-18 after stimulation with one heat-killed isolate of penicillin-susceptible *Streptococcus pneumoniae*; c) interferon-alpha (IFNα) after stimulation with one heat-killed isolate of penicillin-susceptible *Streptococcus pneumoniae*; d) interferon-gamma (IFNγ) after stimulation with phytohemagglutin (PHA); and e) IFNγ after stimulation with one heat-killed isolate of penicillin-susceptible *Streptococcus pneumoniae*. Superscript “a” denotes significant differences compared with healthy volunteers.

In order to define if the effect of H1N1 is related with the production of T_H_-1 type cytokines or not, concentrations of IL-6, IL-18, IFNα and IFNγ were also estimated in supernatants of PBMCs stimulated with either PHA or *Streptococcus pneumoniae* (Figure 4). It was found that stimulation of PBMCs of both patients with flu-like syndrome and H1N1-infection produced greater concentrations of IL-6 compared with healthy volunteers (p: 0.009 for comparisons between flu-like syndrome and healthy volunteers; p: 0.009 for comparisons between H1N1 infection and healthy volunteers). With the exception of single patients, IL-18 and IFNα were below the lower detection limit. Production of IFNγ was greater by PBMCs of H1N1-infected patients after stimulation either with PHA (p: 0.010 compared with healthy volunteers) or with *S.pneumoniae* (p: 0.029 compared with healthy volunteers).

### Effects in the Adaptive Immune System

**Figure 5:**
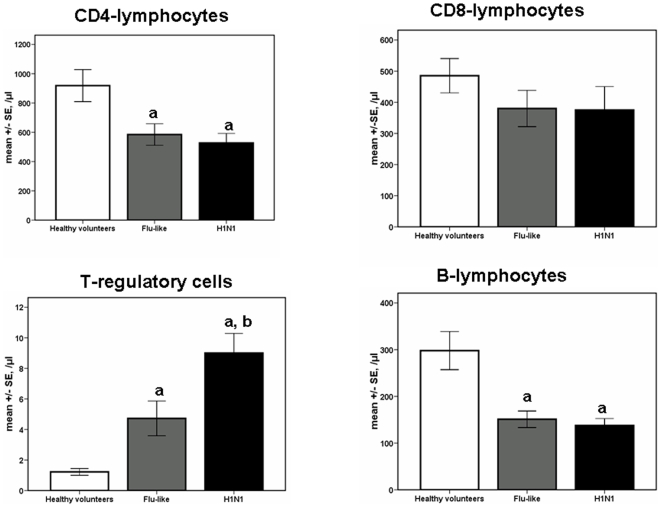
Absolute counts of CD4-lymphocytes, of CD8-lymphocytes, of T-regulatory cells and of B-lymphocytes. Results refer to 10 healthy volunteers, 18 patients with flu-like syndrome and 31 patients infected by the H1N1 virus. a: denotes statistically significant differences compared with healthy volunteers; b: denotes statistically significant differences compared with patients with flu-like syndrome.

Infection by the H1N1 virus was accompanied by significant decrease of CD4-lymphocyte counts (p: 0.003 compared with healthy volunteers) and of B-lymphocyte counts (p<0.0001 compared with healthy volunteers). A significant increase of Tregs was also found compared with healthy volunteers (p: 0.001) (Figure 5).

Mean rates of apoptosis of CD4-lymphocytes were 9.94%, 17.7% and 11.2% among healthy volunteers, among patients with flu-like syndrome and among patients infected by the H1N1 virus respectively (p non-significant between groups). Respective mean rates of apoptosis of B-lymphocytes were 16.2%, 18.4% and 17.3% (p non-significant between groups). Respective mean rates of apoptosis of CD8-lymphocytes were 36.9%, 44.1% and 39.3% (p non-significant between groups).

### Comparisons between H1N1-Infected Patients without and with Pneumonia

**Figure 6:**
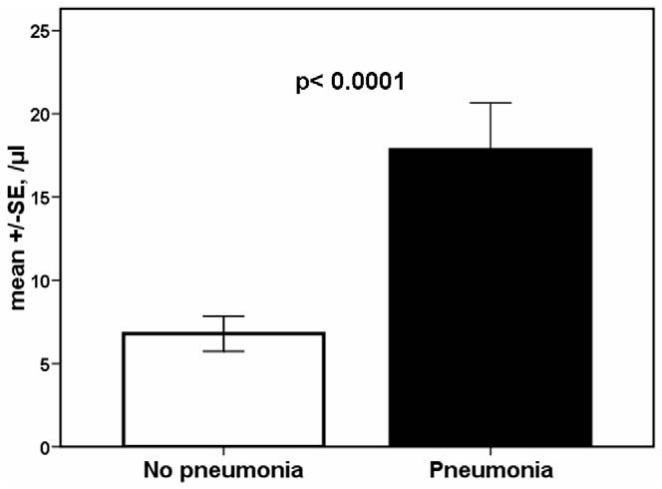
Absolute counts of T-regulatory cells of patients infected with the H1N1 virus. Results are given separately for 25 patients without pneumonia and for six patients with pneumonia (p<0.0001).

Any of the above estimated parameters of the innate and adaptive immune systems were compared between 25 H1N1-infected patients without pneumonia and six patients with H1N1-related pneumonia. No differences were found between them with the sole exception of Tregs counts being greater among the latter compared with the former (p<0.0001) (Figure 6).

### Serum Cytokines

**Figure 7:**
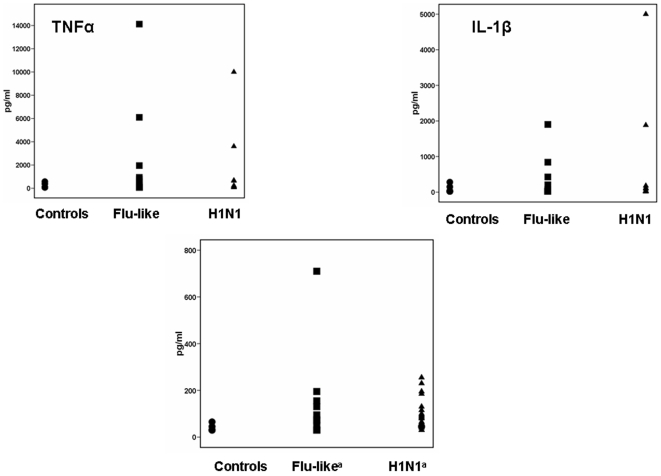
Serum levels of tumour necrosis factor-alpha (TNFα), of interleukin-1beta (IL-1β) and of IL-6. Results refer to 10 healthy volunteers, 18 patients with flu-like syndrome and 31 patients infected by the H1N1 virus enrolled in the study. Superscript “a” denotes significant differences compared with healthy volunteers.

Concentrations of TNFα and IL-1β in serum did not differ between the three groups. Those of IL-6 were higher in serum of patients with flu-like syndrome compared with healthy volunteers (p: 0.025) and in patients with H1N1 infection compared with healthy volunteers (p: 0.034) (Figure 7).

### Over-Time Changes

**Figure 8:**
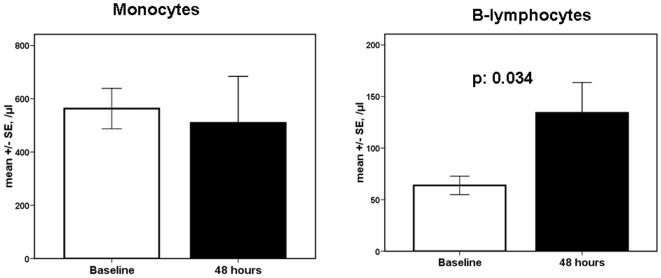
Absolute counts of monocytes and B-lymphocytes. Results refer to 31 patients infected by the H1N1 virus at baseline and after 48 hours. P values denote statistically significant differences between the two time points.

Absolute counts of monocytes and of B-lymphocytes of patients infected by the H1N1 virus at baseline and after 48 hours are shown in Figure 8. Counts of monocytes did not change; however absolute counts of B-lymphocytes were increased (p: 0.034 compared with baseline).

## Discussion

The emerging flu pandemic by the H1N1 virus creates considerable dilemmas in all health-care authorities about the real threat for the human host. It is traditionally been conceived that danger to the host is created when infection by an influenza type A strain predisposes to secondary infections by bacterial pathogens [2]. Estimation of that danger for the public health requires in-depth knowledge of the effects of the emerging H1N1 virus in the innate and adaptive immune responses of the host. To fully elucidate this, immune responses of laboratory-confirmed cases were studied and compared with those of healthy volunteers and those of patients with flu-like syndrome who were negative for infection by H1N1.

Results revealed that within the first two days of advent of the first symptoms, considerable changes of both the innate and the adaptive immune responses were found among patients infected by the H1N1 virus. Principal changes were a) increase of absolute monocyte counts; b) selective defect of TNFα and IFNγ production from PBMCs after stimulation with *S.pneumoniae*; and c) increase of absolute counts of Tregs mainly observed among patients with H1N1-related pneumonia.

Despite the increase of absolute monocyte counts, the expression of HLA-DR on monocytes remained very high. This is of considerable importance for the effect of H1N1 infection on our immune system. High level of expression of HLA-DR on monocytes is an index of the ability of the innate immune system for antigen-presentation. Suppression of the expression of HLA-DR on monocytes is characteristic of immunoparalysis that supervenes in moribund conditions like sepsis and that creates predisposition for super-infection by a variety of pathogens [5]. This does not seem to happen in the event of infection by H1N1 influenza.

To fully clarify the effect of infection by H1N1 on the monocyte function, PBMCs of H1N1-infected patients were stimulated with a variety of stimuli representative of a broad range of pathogens like Gram-negative bacteria, Gram-positive cocci and fungi. Production of cytokines was adequate for most of the applied stimuli being similar to that of healthy volunteers. Pro-inflammatory cytokines were also estimated in serum of patients. IL-6 was increased in both patients with flu-like syndrome and patients with H1N1 infection. In an earlier study of 39 patients with either influenza A or B in Hong Kong during the first semester of 2006 i.e. well before the arrival of H1N1, concentrations of pro-inflammatory cytokines were estimated in plasma and found particularly elevated [6].

Defective cytokines responses were noted after stimulation of PBMCs with PHA and with *S.pneumoniae* and involved production of TNFα and of IFNγ among the panel of estimated cytokines. This implies that infection by H1N1 creates a selective impairment of the innate immune response for *S.pneumoniae* probably mediated through the T_H_1 immune response.

Infection by H1N1 was also accompanied by alterations of the adaptive immune responses. Decreases of CD4-lymphocytes and of B-lymphocytes and increase of Tregs were observed. The former decreases occurred not only in H1N1-infected patients but also in enrolled patients with flu-like syndrome. Apoptosis was not a mechanism involved in the induction of these changes. However, increase of the absolute count of Tregs was a predominant finding of H1N1 infection particularly when infection involved the lower respiratory tract. This may be a sign of an attempt of the host to counterbalance exaggerated immune responses since Tregs are acting by enhancing endogenous anti-inflammatory responses [7].

B-lymphocytes also started to increase within the first 48 hours after baseline counting. This is a very optimistic sign for the impact of H1N1 infection on our health since B-lymphocytes are responsible for antibody production.

However several limitations of this study should be addressed. Viral load of H1N1 and probable correlations with the described immune alterations were not assessed. It should also be underscored that the study was focused on circulatory effector cells whereas potent immune cells may be accumulated in the infection site. Even if this is the case, the validity of the study remains intact since results describe a qualitative derangement of circulatory effector cells within the first 48 hours of initiation of symptoms of infection by the new H1N1 virus.

The presented results revealed that infection by the H1N1 virus is accompanied by a characteristic impairment of the innate immune responses characterized by defective cytokine responses to *S.pneumoniae*. This is accompanied by alterations of the adaptive immune responses predominated by increase of Tregs. The significance of the presented results is underscored by three key-elements: a) alterations of the immune responses are shown even with a study enrolling a relative few number of patients; b) H1N1-infected cases are either of moderate severity or of considerable severity since they are all presented with fever above 38.5°C whereas pneumonia is presented in six; and c) enrolled patients infected by H1N1 influenza are young of a mean age of 28.4 years without any underlying disease. It may be postulated that since impairments of the immune responses for *S.pneumoniae* are even seen for young infected patients, these findings have considerable value for patients with underlying diseases predisposing to pneumococcal infections. It should be further underscored that reported cases of severe infection by the H1N1 virus accompanied by pneumonia and increased mortality were between 15 and 44 years old [8], [9] i.e. within the age range of patients enrolled in the present study. In all cases, the results of the present study may help to better understand the life cycle of H1N1 in the human host and to improve our strategies to control it.

## Methods

### Ethics Statement

The study was performed during the period July-August 2009. The study protocol was approved by the Ethics Committee of the ATTIKON University Hospital of Athens. All patients admitted in the emergency department for flu-like symptoms and who provided written-informed consent were eligible. Only patients with core temperature greater than 38.5°C were further screened for enrolment.

### Study Design

Inclusion criteria were: a) written informed consent; b) age more or equal to 16 years; c) symptoms compatible with infection by H1N1 as already defined [10]; d) core temperature above 38.5°C; and e) start of symptoms within the last two days. Exclusion criteria were: a) deny to consent; b) known infection by the human immunodeficiency virus (HIV); c) neutropenia defined as an absolute neutrophil count equal to or below 500 neutrophils per microliter (µl) of blood; d) oral intake of corticoids defined as more than 1 mg/kg of body weight of equivalent prednisone for more than one month.

Enrolled patients underwent a detailed work-out comprising case-history and epidemiological history, thorough physical examination, chest X-ray if considered necessary, white blood cell count, and urine analysis for detection of leukocytes and nitrate and detection of antigens of *Streptococcus pneumoniae* and of *Legionella pneumophila*. If the above work-out failed to disclose the presence of bacterial infection, a pharyngeal smear was collected with a swab. Infection by the H1N1 virus was diagnosed by the real-time reverse transcriptase polymerase chain reaction detecting the presence of H1N1 RNA in the infected cells (rRT-PCR) [11]. A patient was considered to present with a lower respiratory tract infection (LRTI) due H1N1 virus when a local infiltrate was diagnosed in chest X-ray and H1N1 RNA was detected in the pharyngeal swab, as defined elsewhere [8], [9]. Patients were followed-up until complete resolution of symptoms.

In parallel with the collection of the pharyngeal smear, 25 milliliters of whole blood were collected after venipuncture of one forearm antecubital vein under sterile conditions. Twenty ml blood were collected into two EDTA-coated tubes (Vacutainer, Becton Dickinson, Cockeysville Md) and another 5 were collected into sterile and pyrogen-free tubes (Vacutainer) and transferred immediately to the laboratory for further analysis. Blood was also sampled from 10 healthy volunteers.

For patients diagnosed with H1N1 infection, 5 ml of blood was also sampled after 48 hours as described above.

### Laboratory Procedure

In whole blood collected into the first tube, red blood cells were lyzed with ammonium chloride 1.0 mM. White blood cells were washed three times with phosphate buffered saline (PBS, pH 7.2) (Merck, Darmstadt, Germany) and subsequently incubated for 15 minutes in the dark with the monoclonal antibodies anti-CD3, anti-CD4, anti-CD14 and anti-CD19 and the protein ANNEXIN-V at the flurochrome fluorescein isothiocyanate (FITC, emission 525 nm, Immunotech, Marseille, France); with the monoclonal antibodies anti-CD4, anti-CD8, anti-CD14, anti-CD(16+56), anti-CD25 and anti-HLA-DR at the fluorochrome phycoerythrin (PE, emission 575 nm, Immunotech); with the monoclonal antibody anti-CD3 and anti-CD127 at the fluorochrome ECD (emission 613 nm, Immunotech); with the monoclonal antibody anti-CD45 at the fluorochrome PC5 (emission 650 nm, Immunotech); and with 7-AAD at the fluorocolour PC7 (emission 670 nm, Immunotech). Fluorospheres (Immunotech) were used for the determination of absolute counts. The following combinations were studied: anti-CD3/anti-CD4/anti-CD45 for CD4-lymphocytes; anti-CD3/anti-CD8/anti-CD45 for CD8-lymphocytes; anti-CD3/anti-CD(16+56)/anti-CD45 for Natural Killer (NK)-cells and for NKT-cells; anti-CD4-/anti-CD25/anti-CD127/anti-CD45 for T-regulatory cells (Tregs); anti-CD19/anti-CD45 for B-lymphocytes; anti-CD14/anti-HLA-DR/anti-CD45 for immunoparalysis of monocytes; ANNEXIN-V/anti-CD4/anti-CD3/7-AAD for apoptosis of CD4-lymphocytes; ANNEXIN-V/anti-CD8/anti-CD3 for apoptosis of CD8-lymphocytes; ANNEXIN-V/antiCD14/7-AAD for apoptosis of monocytes; ANNEXIN-V/anti-CD19/7-AAD for apoptosis of B-lymphocytes. Cells were analyzed after running through the EPICS XL/MSL flow cytometer (Beckman Coulter Co, Miami, Florida) with gating for lymphocytes or monocytes based on their characteristic FS/SS scattering. Cells staining negative for CD3 and positive for CD(16+56) were considered NK-cells; those staining positive for both CD3 and CD(16+56) were considered NKT- cells. Cells staining positive for both CD4 and CD25 and negative for CD127 were considered Tregs, according to other authors [12], [13]. In all analyzed cell subtypes, apoptotic cells stained positive for ANNEXIN-V and negative for 7-AAD. Isotypic IgG controls were used for each patient.

To verify the appropriateness to discriminate cells staining positive for both CD4 and CD25 and negative for CD127 as Tregs, blood sampling was repeated for seven newly enrolled patients; two healthy volunteers, two patients with flu-like syndrome and three patients with H1N1 infection. Absolute counts of Tregs were estimated both as described above and with the PE anti-human Foxp3 staining set (eBioscience Inc., San Diego, USA). Median difference of the two assays was 5.88%.

Peripheral blood mononuclear cells (PBMCs) were isolated from blood collected in the second tube after gradient centrifugation over Ficoll (Biochrom, Berlin, Germany) for 20 minutes at 1400 g. After three washings in ice-cold PBS pH 7.2, PBMCs were counted in a Neubauer plate with trypan blue exclusion of dead cells. They were then diluted in RPMI 1640 enriched with 2 mM of L-glutamine, 500 µg/ml of gentamicin, 100 U/ml of penicillin G and 10 mM of pyruvate (Biochrom) and suspended in wells of a 96-well plate. The final volume per well was 200 µl with a density of 2×10^6^ cells/ml.

PBMCs were exposed in duplicate to the following stimuli for 24 hours at 37°C in 5% CO_2_: 10 ng/ml of *Escherichia coli* O55:B5 lipopolysaccharide (LPS, Sigma, St. Louis, USA); 5 µg/ml of phytohemagglutin (PHA, EMC microcolecctions, Tübingen, Germany); and 2×10^5^ colony forming units/ml of each of the following heat-killed isolates: *Candida albicans*; *Pseudomonas aeruginosa*; methicillin-resistant *Staphylococcus aureus* (MRSA) producing Panton-Valentine leukocidin (PVL); and penicillin-susceptible *S.pneumoniae* (PSSP). All the above were clinical isolates derived from the blood of different patients with documented infections. Resistance of *S.aureus* to methicillin and production of PVL were documented by the detection of the *mec*A gene and the *luc*S-PV gene respectively by PCR [14], [15]. PSSP has been isolated from a patient with LRTI and bacteremia and it has already been described in a previous study [16].

Concentrations of tumour necrosis factor-alpha (TNFα) and of interleukin-1beta (IL-1β) were estimated in cell supernatants in duplicate by an enzyme immunoassay (R&D, Minneapolis, USA). Those of interferon-gamma (IFNγ) were estimated only in supernatants of PHA-stimulated cells and of PSSP-stimulated cells (Diaclone, Paris, France). Those of IL-6, of IL-18 and of IFNα were estimated only in supernatants of PSSP-stimulated cells (Diaclone, Paris, France and MBL, Nagoya, Japan). The lowest detections limits were: for TNFα 80 pg/ml; for IL-1β 20 pg/ml; for IL-6 60 pg/ml; for IL-18 130 pg/ml; for IFNα 40 pg/ml; and for 100 IFNγ pg/ml.

Blood collected into sterile and pyrogen-free tubes was centrifuged and serum was stored at −70°C until assayed. Concentrations of TNFα, IL-1β and IL-6 were estimated as described above.

In blood samples collected 48 hours after the baseline, absolute counts of monocytes and of B-lymphocytes were determined as described above.

### Statistical Analysis

Subjects were divided into healthy volunteers; into those presenting with flu-like syndrome; and into those infected by the H1N1 virus. Results were expressed as means ± SE. Comparisons between groups were compared by one-way analysis of variance (ANOVA) with post-hoc Bonferroni analysis for the avoidance of random correlations. Comparisons between H1N1-infected patients without and with pneumonia were done by Student's “t-test”. Comparisons between consecutive samplings were done by paired “t-test”. Any value of p below 0.05 was considered significant.
